# Adult-Onset Multisystem Langerhans Cell Histiocytosis: Atypical Skeletal and Endocrine Manifestations

**DOI:** 10.7759/cureus.107356

**Published:** 2026-04-19

**Authors:** Philipp Hacker, Christian Benignus, Leonie Frauenfeld, Frank Traub

**Affiliations:** 1 Department of Orthopaedic Surgery, University Hospital Tübingen, Tübingen, DEU; 2 Department of Anaesthesiology, University Hospital Tübingen, Tübingen, DEU; 3 Department of Pathology, University Hospital Tübingen, Tübingen, DEU; 4 Department of Orthopaedics and Traumatology, University Medical Center of the Johannes Gutenberg University Mainz, Mainz, DEU

**Keywords:** adult-onset lch, bone lesions, central diabetes insipidus, differential diagnosis, langerhans cell histiocytosis (lch), multisystem disease, systemic therapy

## Abstract

Langerhans cell histiocytosis (LCH) is a rare clonal histiocytic neoplasm that is uncommon in adults and may be diagnostically challenging because of its heterogeneous clinical presentation. We report a case of a 55-year-old male who presented with persistent right ankle pain and multifocal lytic lesions involving the distal tibia, fibula, talus, calcaneus, and cuboid. His medical history was notable for a pituitary lesion with central diabetes insipidus diagnosed two years earlier.

Open biopsy of the tibia demonstrated a mixed infiltrate containing characteristic Langerhans cells with grooved nuclei and numerous eosinophils. Immunohistochemistry showed strong positivity for CD1a, S100, and langerin (CD207), confirming LCH. Staging studies revealed pituitary involvement and mild radiologic splenomegaly, without evidence of hematopoietic or hepatic risk-organ dysfunction. Retrospective molecular testing on stored tissue was negative for BRAF V600E.

The patient was treated with vinblastine and prednisolone, along with desmopressin and somatotropin replacement. Zoledronic acid was added for osseous disease. Over 70 months of follow-up, the patient achieved durable clinical and radiologic disease control, with pain improving from 7/10 to 1/10 and no evidence of relapse at last contact. Treatment-related toxicity was limited to mild peripheral neuropathy.

This case highlights an unusual presentation of adult LCH with distal lower-extremity and tarsal bone involvement, as well as hypothalamic-pituitary disease preceding osseous diagnosis by two years. It underscores the importance of considering LCH in adults with unexplained multifocal lytic bone lesions and endocrine dysfunction, and it demonstrates that durable disease control can be achieved with systemic therapy.

## Introduction

Langerhans cell histiocytosis (LCH) is a rare clonal proliferative disorder characterized by the accumulation of Langerhans-type dendritic cells in various organs. Although more commonly diagnosed in children, the estimated incidence in adults is approximately 1.8 per million, with a mean age of 34 years [1,2]. The current literature suggests a slight male predominance or equal gender distribution [3].

In adults, the most frequently affected organs are the bones (77%) and the skin (39%). Central nervous system (CNS) involvement occurs in approximately 6% of cases and commonly affects the pituitary gland, resulting in central diabetes insipidus (DI) or other endocrine abnormalities [4,5]. Osseous lesions typically occur in the skull, ribs, pelvis, and diaphyseal regions of long bones. In contrast, involvement of the epiphysis, metaphysis, or small bones of the hands and feet is rare [6].

The clinical manifestations of LCH vary widely and often mimic other conditions, including infections, inflammatory disorders, benign bone tumors, and malignancies such as lymphoma. Therefore, diagnosis requires a combination of clinical, radiological, and histopathological evaluations. A definitive diagnosis relies on identifying characteristic histological and immunohistochemical markers, such as CD1a, S100, and langerin [6,7].

Here, we describe an unusual case of adult multisystem LCH presenting with multiple lytic bone lesions in the distal lower extremities and feet, accompanied by pituitary gland involvement. This case is particularly instructive because the patient’s pituitary lesion and endocrine dysfunction preceded the osseous diagnosis by two years, delaying recognition of multisystem LCH. The rare distribution of lytic lesions in the distal lower extremities and tarsal bones, together with significant overlap with infectious, inflammatory, neoplastic, and other histiocytic disorders, underscores the importance of a structured diagnostic workup and timely systemic treatment.

## Case presentation

A 55-year-old Caucasian male presented in 2016 to the orthopedic outpatient clinic with a four-month history of progressively worsening right ankle pain. The symptoms began shortly after a minor motor vehicle accident and were described as load-dependent, without nocturnal worsening or rest pain. He denied fever, weight loss, neurological complaints, or other systemic symptoms.

The medical history was notable for post-thrombotic syndrome in both lower extremities, a previous erysipelas infection of the right leg, obstructive sleep apnea, morbid obesity (BMI = 41.6 kg/m²), arterial hypertension, and impaired glucose tolerance. A pituitary lesion had been identified two years before the onset of the ankle symptoms and was under endocrine surveillance. MRI at that time demonstrated loss of the posterior pituitary bright spot and thickening of the infundibulum. Central diabetes insipidus had already been diagnosed and treated as presumed idiopathic CDI. A biopsy was not pursued because no extracranial lesion was present, and a pituitary biopsy was considered disproportionate. In retrospect, these findings were considered the first clinical manifestation of LCH. The patient had no history of malignancy, chronic infection, or autoimmune disease.

Physical examination revealed mild tenderness on palpation above the right ankle joint without swelling, erythema, deformity, or movement restriction. The gait was normal, and the adjacent joints were unremarkable.

Imaging

**Figure 1:**
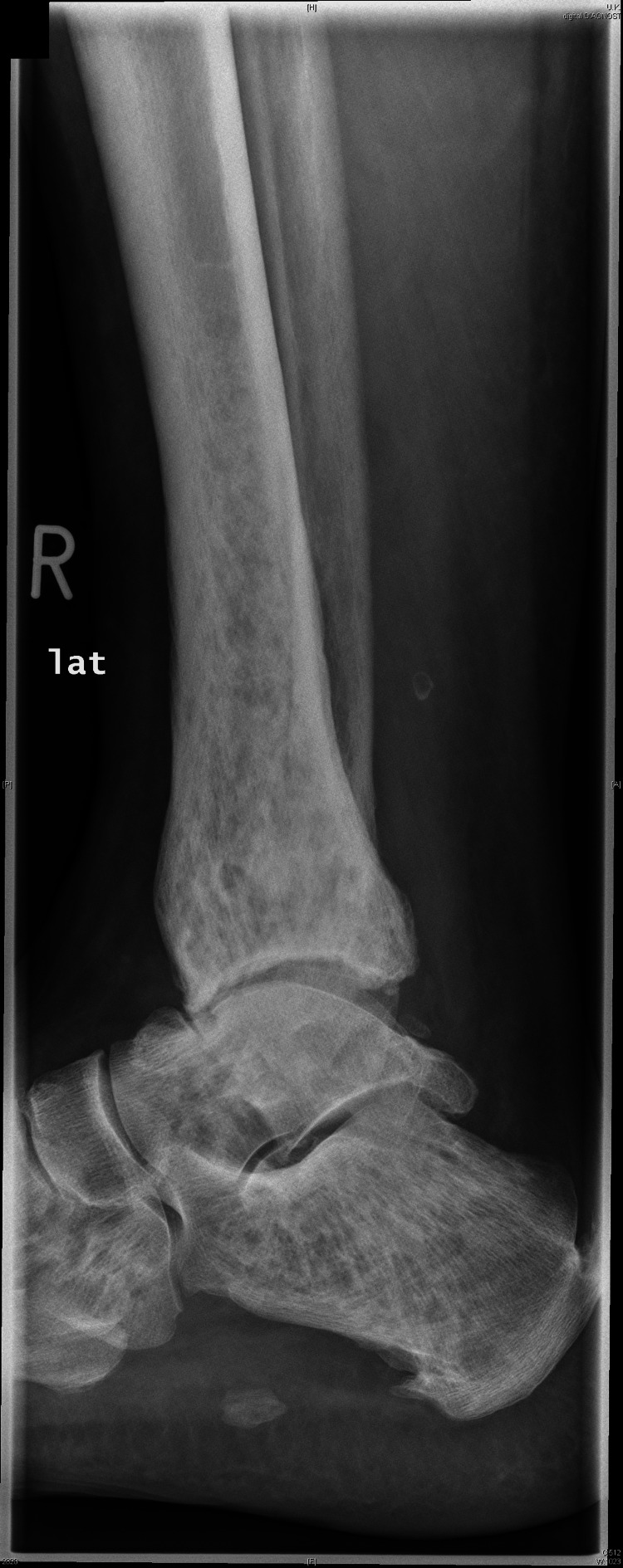
A lateral view of the right tibia, talus, and calcaneus in a conventional X-ray showed small, spotted, and partially confluent areas.

Initial radiography revealed small, spotted, partially confluent areas of altered density in the tibia, fibula, talus, and calcaneus (Figure 1).

**Figure 2:**
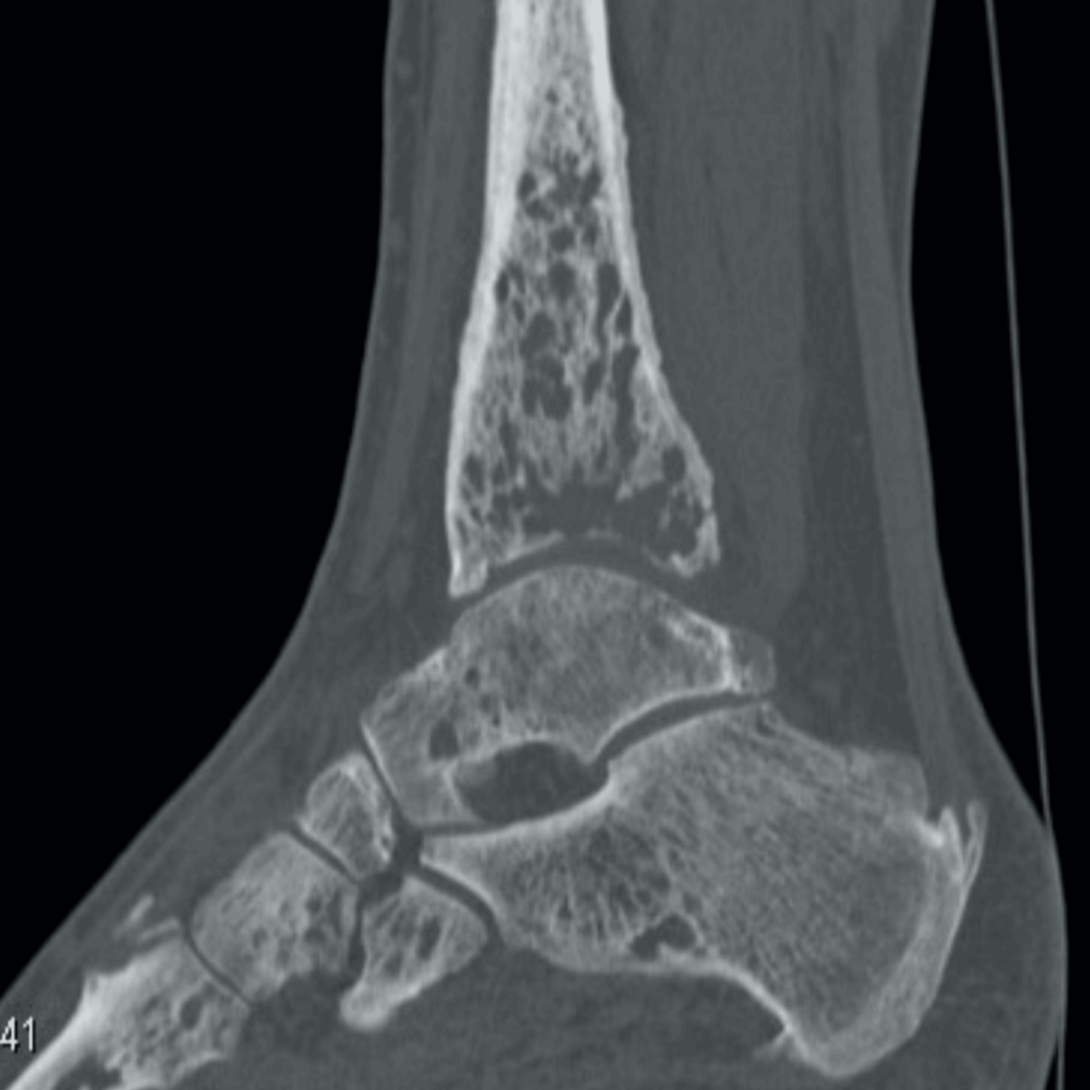
Lateral view of the right foot and tibia on CT.

Computed tomography (CT) revealed additional lytic lesions in the cuboid, talus, and calcaneus of the right foot, in addition to those identified in the tibia (Figure 2).

**Figure 3:**
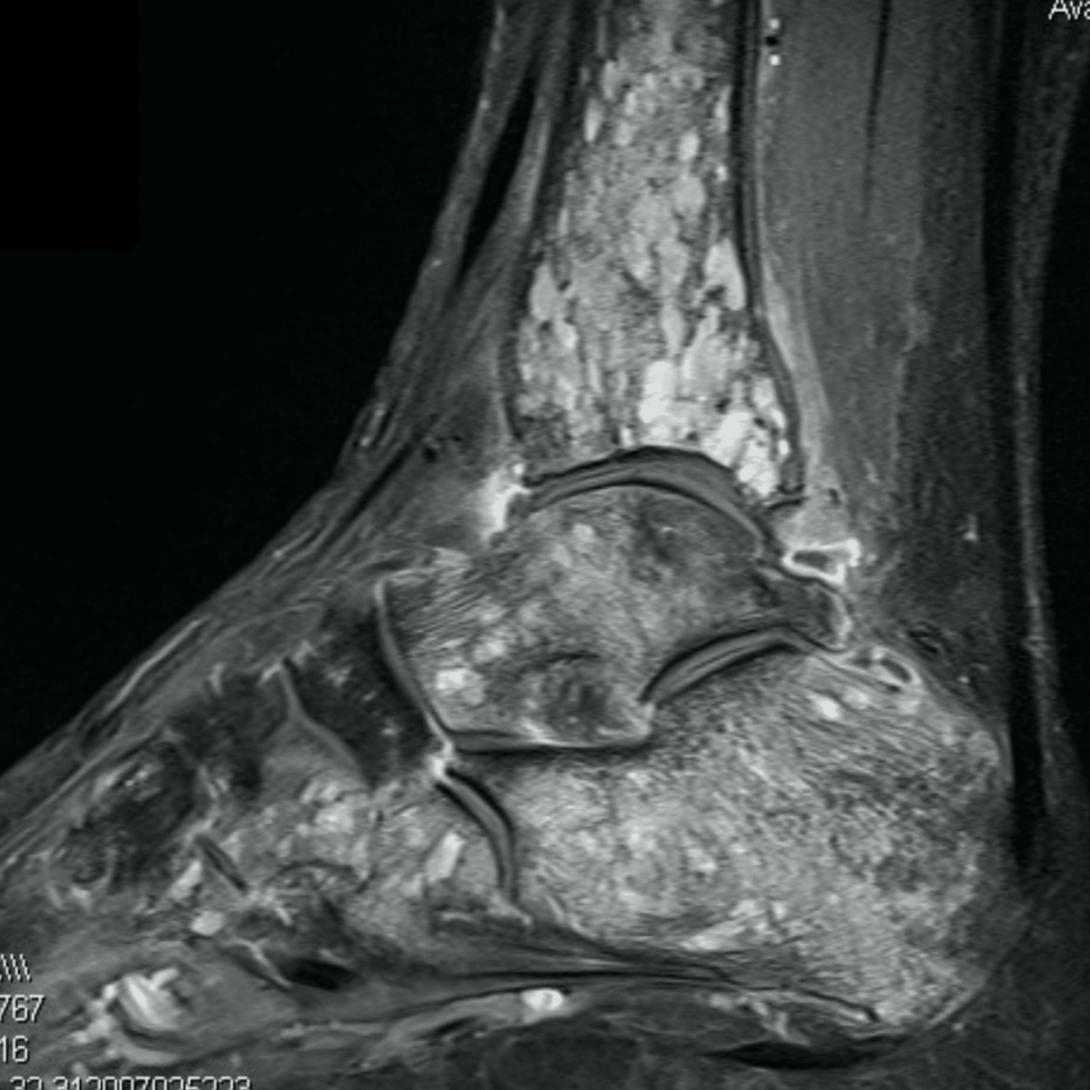
Sagittal contrast-enhanced MRI (gadolinium) showed multiple additional lesions in the hindfoot and tarsal bones, in addition to the previously identified lesions.

**Figure 4:**
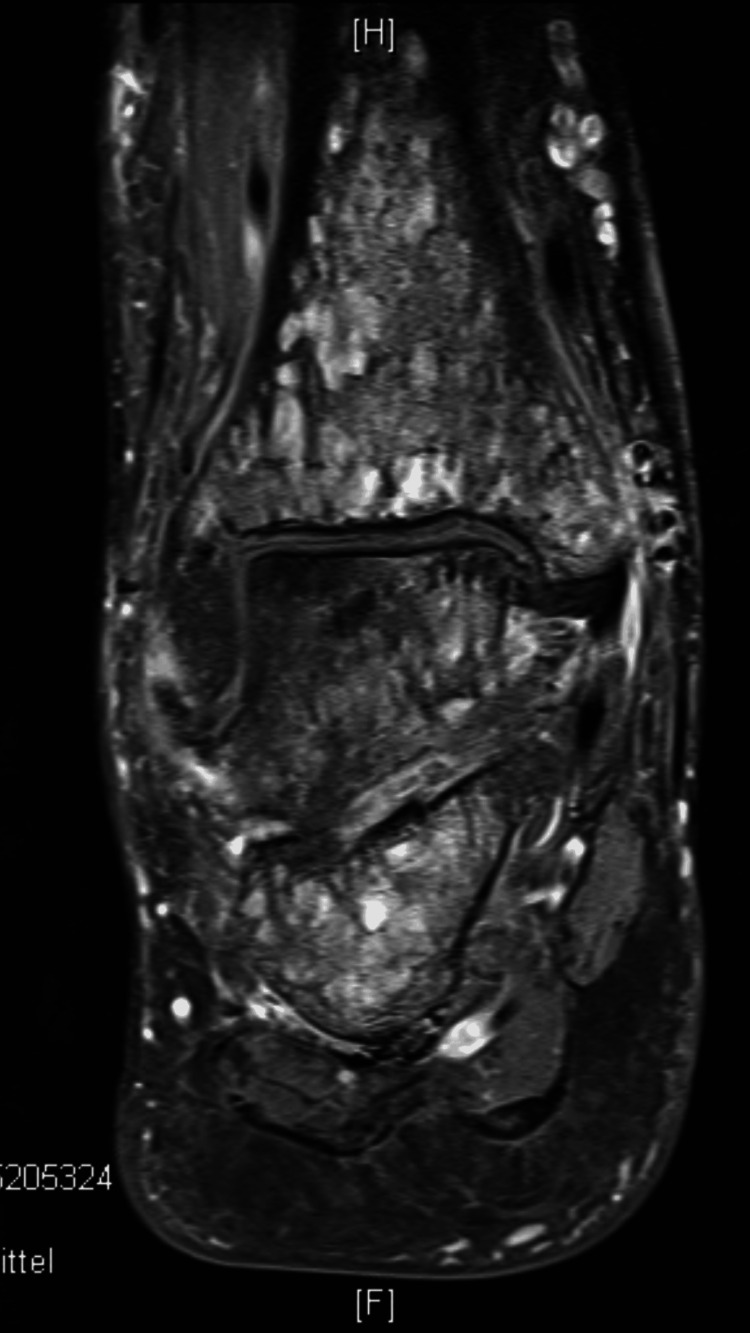
Coronal gadolinium-enhanced MRI demonstrated multiple lesions involving the tibia, talus, and calcaneus.

Gadolinium-enhanced MRI confirmed and further characterized these lesions. The lesions were isointense to muscle on T1-weighted sequences and hyperintense on T2-weighted sequences, with associated surrounding bone marrow edema (Figures 3, 4).

Given the multifocal distribution and absence of an aggressive periosteal reaction, a systemic infiltrative disorder was considered more likely than a primary bone sarcoma.

Diagnostic workup

Laboratory investigations revealed only mild elevation of inflammatory markers, with a C-reactive protein (CRP) level of 8.2 mg/L and an erythrocyte sedimentation rate (ESR) of 23 mm/h. The leukocyte count was slightly elevated at 11,200/µL. These nonspecific findings made an acute high-grade infection less likely but did not rule out chronic infections. Endocrine evaluation demonstrated hypernatremia (serum sodium = 148 mmol/L), elevated plasma osmolality (315 mOsm/kg), and inappropriately low urine osmolality (280 mOsm/kg), with a positive response to desmopressin, confirming central diabetes insipidus. Serum insulin-like growth factor 1 (IGF-1) was 58 ng/mL, which was low for age according to the local laboratory reference range. Adult growth hormone (GH) deficiency was confirmed by an insulin tolerance test, showing an inadequate peak GH response. The adrenal, thyroid, and gonadal axes, as well as prolactin, were within normal limits.

An open biopsy of the right tibia was performed. Histopathological examination revealed a mixed cellular infiltrate characterized by Langerhans cells with oval to reniform nuclei, longitudinal grooves ("coffee-bean" appearance), inconspicuous nucleoli, and moderate eosinophilic cytoplasm, admixed with numerous eosinophils and scattered lymphohistiocytic inflammatory cells. Immunohistochemistry confirmed the diagnosis of LCH, with strong and diffuse positivity for CD1a, S100, and langerin (CD207). CD68 and CD163 were also positive, while CD99 and pan-cytokeratin were negative. This profile definitively established the diagnosis of LCH. Furthermore, retrospective molecular profiling was performed on stored formalin-fixed paraffin-embedded (FFPE) tissue. BRAF V600E was not detected in three separate runs, indicating a BRAF wild-type case.

Staging studies, including whole-body PET/CT, revealed mild splenomegaly (measured 15 cm in longitudinal diameter). In the absence of cytopenia, liver dysfunction, or other clinical evidence of splenic compromise, this finding was interpreted as isolated radiologic splenomegaly without clear evidence of functional risk-organ involvement. No pulmonary or cutaneous lesions were detected. Cranial magnetic resonance imaging (MRI) revealed pituitary gland infiltration.

Taken together, these findings established the diagnosis of multisystem LCH involving bone and the hypothalamic-pituitary axis, without hematopoietic or hepatic dysfunction.

Treatment

The patient began systemic therapy in 2016, consisting of vinblastine (6 mg/m² weekly for six weeks) combined with prednisolone (40 mg/m² weekly for four weeks, tapered over two weeks). Endocrine insufficiency was managed with desmopressin and somatotropin. After six treatment cycles, follow-up PET/CT and cranial MRI revealed stable lesions with partial symptomatic improvement. Maintenance therapy with vinblastine (6 mg/m² on day one) and prednisolone (4 mg/m² on days one to five) was administered every three to four weeks for 12 months. Bisphosphonate therapy with zoledronic acid (4 mg every 12 weeks) was administered due to bone involvement.

Outcome and follow-up

The patient was followed for 70 months after treatment initiation. Serial imaging (MRI and PET/CT) performed throughout this period demonstrated sustained disease stability, with mild regression of the lytic lesions in the distal tibia and tarsal bones. The patient remained symptomatically improved, from a visual analog scale score of 7/10 at presentation to 1/10 after treatment. This improvement was accompanied by restoration of comfortable weight-bearing and marked improvement in daily ambulation. Following the first year of treatment, zoledronic acid was continued as an annual maintenance dose for five subsequent years. Endocrine status remained stable under hormone replacement. After the 70-month follow-up visit, the patient was lost to further follow-up. At the final point of contact, there was no clinical or radiological evidence of disease relapse or progression.

## Discussion

This case illustrates two features that are unusual in adult LCH: involvement of multiple distal lower-extremity and tarsal bones, and a hypothalamic-pituitary manifestation that preceded osseous diagnosis by two years. Together, these features complicated the diagnostic pathway and broadened the differential diagnosis substantially [1].

The radiological findings in this case required careful evaluation. Multifocal lytic bone lesions can mimic a variety of conditions, including osteomyelitis, hyperparathyroidism, Paget’s disease, benign bone tumors (e.g., enchondromas and giant cell tumors), and malignant tumors such as lymphoma or metastasis [6,8]. Additionally, the imaging pattern raised the possibility of Erdheim-Chester disease (ECD), which can also affect the long bones, often in a symmetric juxta-articular distribution [9]. In our patient, the presence of multiple distal limb lesions and pituitary involvement was suggestive of ECD, which typically shows a CD68+/CD163+ but CD1a−/langerin− phenotype. In contrast, the lesional cells in this case showed strong positivity for CD1a, S100, and langerin, supporting LCH and excluding classic ECD. Thus, biopsy remains indispensable for confirming the diagnosis and distinguishing LCH from other similar histiocytic disorders. This was particularly important in the present case because the combination of distal long-bone/foot lesions and pituitary involvement could easily have suggested an alternative histiocytic neoplasm on imaging grounds alone.

At the time of initial diagnosis in 2016, molecular profiling for BRAF V600E mutations was not yet routinely integrated into the clinical diagnostic workflow for adult LCH at our center. However, in accordance with current international consensus, retrospective molecular analysis was performed on stored FFPE tissue. BRAF V600E was not detected in three separate runs, indicating a BRAF wild-type case. This finding is of clinical interest, as BRAF status may have prognostic relevance in adult LCH; however, in the present case, no firm causal inference can be drawn between BRAF wild-type status and the observed long-term disease stability.

Endocrine abnormalities, particularly central diabetes insipidus (DI), are common in adult LCH with CNS involvement [4]. DI may precede or follow the onset of bone lesions. Growth hormone deficiency (GHD), though clinically nonspecific and often underrecognized, was also identified in this patient, confirming multisystemic disease [5]. The presence of such endocrine dysfunction in patients with unexplained bone pain should prompt consideration of multisystem histiocytic disease and appropriate systemic evaluation.

There is no universally accepted standard therapy for adult LCH due to its low incidence and variability in presentation. Curettage may be curative for patients with solitary bone lesions [8]. However, systemic therapy is indicated for multisystemic or multifocal bone disease [10]. Historically, treatment regimens have been extrapolated from pediatric protocols, establishing the combination of vinblastine and prednisolone as a widely used, first-line therapy. While some recent expert consensus guidelines favor cytarabine for adults to minimize the risk of vinca-alkaloid-associated neurotoxicity, vinblastine/prednisolone remains a recommended evidence-based option, particularly for multisystem disease [1,11].

In the present case, this regimen was selected and was associated with durable disease control. Treatment was well tolerated, causing only mild, grade 1 peripheral neuropathy [12]. The addition of bisphosphonates has been explored in LCH with bone involvement to reduce pain and possibly modulate osteoclast activity; our patient’s 70-month progression-free survival suggests that this combined approach can achieve durable disease control in selected adults with multisystem disease [10,13].

This case is notable for several reasons: (1) the extensive involvement of multiple small bones of the foot and distal limb, which is rarely reported in adults; (2) hypothalamic-pituitary disease with central diabetes insipidus preceding osseous diagnosis by two years; (3) durable long-term disease control over more than 70 months after vinblastine/prednisolone plus bisphosphonate therapy.

Overall, this case emphasizes the diagnostic importance of integrating atypical skeletal findings with endocrine abnormalities and demonstrates that durable control is achievable even in adult multisystem disease with an unusual presentation.

## Conclusions

Adult LCH should be considered in the differential diagnosis of unexplained multifocal lytic bone lesions, particularly when the distribution is atypical and accompanied by endocrine abnormalities such as central diabetes insipidus. This case emphasizes that hypothalamic-pituitary involvement may precede osseous disease by years, delaying recognition of multisystem LCH. Because imaging findings overlap with those of infection, malignancy, and other histiocytic disorders, histopathologic confirmation with appropriate immunohistochemistry remains essential. In the present patient, vinblastine/prednisolone combined with bisphosphonate therapy achieved durable long-term clinical and radiologic disease control.
